# Determination of Potential Therapeutic Targets and Prognostic Markers of Ovarian Cancer by Bioinformatics Analysis

**DOI:** 10.1155/2021/8883800

**Published:** 2021-03-19

**Authors:** Jing Zhang, Shouguo Huang, Lini Quan, Qiu Meng, Haiyan Wang, Jie Wang, Jin Chen

**Affiliations:** Department of Gynecology, Affiliated Haikou Hospital of Xiangya Medical College, Central South University, Haikou 570208, Hainan Province, China

## Abstract

This study is to study the expression of CXCRs in ovarian cancer tissues and their value in prognosis. The expressions of CXCR1-CXCR7 mRNA between ovarian tumor tissues and normal tissues and in different pathological types of ovarian tumor tissues were compared by ONCOMINE online tool. The relationship between the expression of CXCRs and clinical pathological staging was studied by GEPIA. Kaplan-Meier plotter online tool was used to analyze prognosis. Finally, GO and KEGG analyses and protein interaction network analysis were performed for CXCRs by the DAVID software to predict their function, and cBioPortal was used to identify the key functional genes. The expression of CXCR3/4/7 mRNA in ovarian cancer tissues was higher than that in normal ovarian tissues, and the expression of CXCR4 was the highest (fold change = 306.413, *P* < 0.05). The expression of CXCR1/2/3/4/7 mRNA in different pathological types of ovarian tumors was significantly different (*P* < 0.05). Only CXCR5 expression level was associated with tumor staging. Survival analysis showed that high CXCR7 mRNA expression and low CXCR5/6 expression were associated with the shortening of overall survival. High CXCR4/7 expression and low CXCR5/6 expression were associated with the shortening of progression-free survival. High CXCR2/4 expression and low CXCR5/6 expression were closely related to the shortening of postprogressing survival. Protein interaction network analysis showed that GNB1, PTK2, MAPK1, PIK3CA, GNB4, GNA11, KNG1, and ARNT proteins were closely related to the CXC receptor family. CXCR3/4/7 are potential therapeutic targets, and CXCR2/4/5/6/7 are new markers for the prognosis of ovarian cancer.

## 1. Introduction

Ovarian cancer is one of the three major gynecological tumors. Compared with other gynecological malignancies, the disease is concealed and lacks early diagnosis methods [1]. Studies have shown that more than 75% of ovarian cancer patients were diagnosed at the advanced stage with extensive tumor spread [2, 3]. Ovarian epithelial malignant tumors account for nearly 90% of all cases of ovarian malignancies [4]. At present, the main treatment method is ovarian cytoreductive surgery plus paclitaxel and platinum-based first-line chemotherapy. However, secondary drug resistance often occurs. The recurrence rate of ovarian cancer is about 70% [5]. The five-year survival rate is about 35% [6], and the 10-year survival rate of high-grade ovarian tumor is less than 15% [7], seriously affecting the prognosis. Drug resistance is the biggest obstacle to the chemotherapy of ovarian cancer and also the main factor affecting the survival of patients [8]. However, how to effectively predict the progress of cancer and the occurrence of drug resistance, implement accurate medical treatment, and identify reliable predictive biomarkers remain to be investigated.

Chemokine receptors (CXCRs) are a class of G-protein-coupled receptors, which are rich in seven transmembrane motifs composed of hydrophobic amino acids. When CXCR binds to its ligand, it activates G protein, which further mediates signal transduction and plays roles in cell growth, division, energy metabolism, phagocytosis, migration, secretion, etc. [9].

CXCRs are classified into CCR, CXCR, XCR, and CX3CR subfamilies according to their ligands. The CXCR subfamily contains 7 receptors of CXCR1, CXCR2, CXCR3, CXCR4, CXCR5, CXCR6, and CXCR7. In recent years, studies have confirmed that some receptors in the CXCR family are closely related to tumor metabolism, immunity, and drug resistance [10–14], and their expression levels can be used as predictors of tumor metastasis and chemotherapy response. However, the activation or inhibition mechanism of the CXCR family in ovarian cancer has not been fully elucidated.

Herein, bioinformatics analysis was used to analyze the possibility of CXCR family members as predictive markers of ovarian cancer occurrence, metastasis, and chemotherapy-sensitivity and prognosis.

## 2. Materials and Methods

### 2.1. ONCOMINE Analysis

The levels of CXCR1-CXCR7 mRNAs in different ovarian cancer tissues, normal tissues, and pathological types of ovarian cancer tissues were analyzed with ONCOMINE. The *t*-test was used. The genes with *P* < 0.05, fold change > 1.5, and in Top10% of gene rank were screened out.

### 2.2. Gene Expression Profile Interaction Analysis (GEPIA) Data Set

GEPIA is an interactive web application based on TCGA and GTEx large genome database data mining and gene function analysis and is an important data analysis tool [15]. On the GEPIA website (http://gepia.cancer-pku.cn/), the expression analysis, correlation analysis, and survival analysis of CXCR genes were performed, and the plots were automatically generated online.

### 2.3. Kaplan-Meier Plotter

The survival curves were plotted using the online Kaplan-Meier plotter database. The overall survival (OS), progression-free survival (PFS), and postprogressive survival (PPS) of ovarian cancer patients were calculated, and the difference in survival was analyzed by logrank test [16]. The cut-off values were determined based on the optimal values of the ROC curve analysis. Statistical analysis and the highest quality calculations were all performed online in the database. The 95% confidence interval (CI) and logrank *P* values were marked on the top right of the figure.

### 2.4. cBioPortal

Using the online database cBioPortal (http://www.cbioportal), the molecular profile changes of the CXCR family in ovarian cancer tissues were calculated, including mutations, putative copy-number alteration from GISTIC, mRNA expression *Z* scores, and RSEM (batch normalized from Illumina HiSeq-RNASeqV.2). The coexpression level was calculated, and the coexpression network map was plotted.

### 2.5. DAVID

Gene Ontology (GO) and KEGG signaling pathway enrichment analyses were performed for the target genes of CXCR family using the DAVID (https://david.ncifcrf.gov/) database to annotate the functions and analyze the biological processes and the mainly involved tumor-related signaling pathways of CXCRs.

### 2.6. Sample Collection

The paraffin-embedded ovarian epithelial malignant tumor tissues were collected from ovarian cancer patients (*n* = 20) from the Department of Pathology, Haikou Hospital, Xiangya School of Medicine, Central South University from January 2014 to September 2017. All patients were not treated with radiotherapy, chemotherapy, hormones, or biological therapy before surgery, and their clinical and pathological information was complete. For control, paraffin-embedded normal ovarian epithelial tissues were collected from 20 cases of patients with ovarian cyst from the same period.

### 2.7. Immunohistochemistry

All paraffin specimens were serially sectioned into 4 *μ*m sections, routinely dewaxed and hydrated. The antigen retrieval was performed. The endogenous peroxidase activity was inactivated by H_2_O_2_. The sections were blocked with rabbit serum and incubated with rabbit anti-human CXCR1-7 polyclonal antibodies (Abcam; CXCR1 (ab137351), CXCR2 (ab65968), CXCR3 (ab133420), CXCR4 (ab74012), CXCR5 (ab203212), CXCR6 (ab8023), and CXCR7 (ab72100)) at 4°C overnight. After washing with PBS, the anti-rabbit secondary antibody was added and incubated for 1 h. Hematoxylin was added for counter staining and then sealed with neutral gum. The images were observed under Olympus 600 autobiochemical analyzer (Tokyo, Japan).

## 3. Results

### 3.1. Expression of CXCR mRNA in Ovarian Tumor and Normal Ovarian Tissues

**Figure 1:**
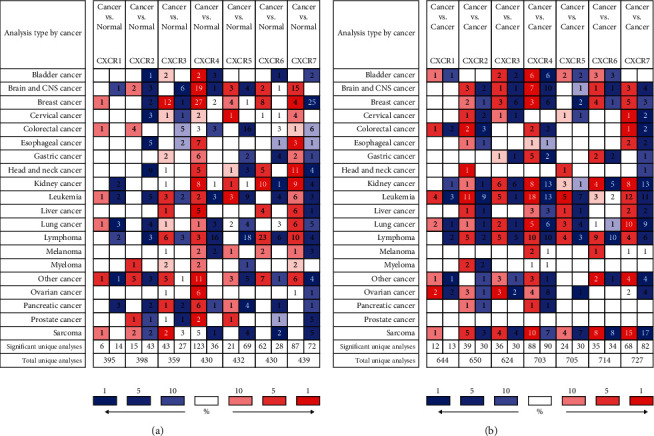
The changes of CXCR mRNA expression between different types of cancer and normal tissues using the ONCOMINE database. (a) Differential expression of CXCR mRNA between various tumor tissues and normal tissues. (b) Differential expression of CXCR mRNA in different pathological types in various tumor tissues. Color of the boxes is determined by the best gene rank percentile for the analysis within the cell, in which red indicates the copy gain, blue indicates the copy loss, and white indicates that the copy number is neutral. The data in the middle of the square represents the number of data sets.

The ONCOMINE database was used to compare the transcription levels of the CXCR family between ovarian tumor and normal ovarian tissues and between different pathological types of ovarian cancer. The results showed that the expression of CXCR3, CXCR4, and CXCR7 mRNA in ovarian cancer tissues was significantly higher than that in normal ovarian tissues (*P* < 0.05). Among them, CXCR4 had the highest expression change (fold change = 306.413, *P* < 0.05), and 6 data sets confirmed this [17–21] (Figure 1(a) and Table 1).

CXCR1 and CXCR2 were downregulated in ovarian cancer tissues, while CXCR5 and CXCR6 were not significantly different between ovarian cancer and normal tissues. Among different pathological types of ovarian cancers, there were various degrees of expression differences, except for CXCR5 and CXCR6, and the gold change ranged from 1.628 to 2.824 (Figure 1(b) and Table 1). The biggest expression difference was found in adenocarcinoma [22]. These results suggest that the expression of CXCR3/4/7 mRNA in ovarian cancer tissues was higher than that in normal ovarian tissues, and there were also differences in ovarian cancer tissues of different pathological types.

### 3.2. Relationship between CXCR mRNA Levels and Clinical Stage of Ovarian Cancer

**Figure 2:**
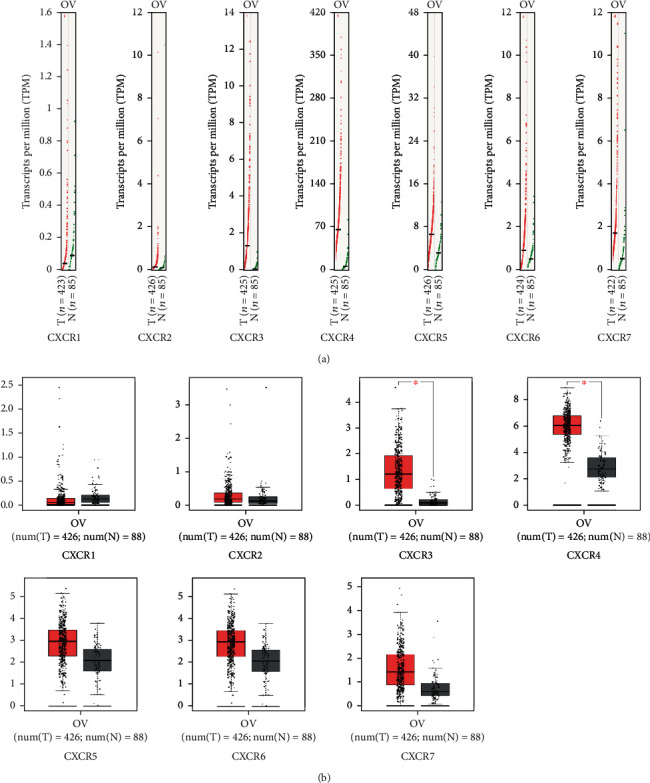
The expression of CXCRs in ovarian cancer (GEPIA). (a) Differential expression of CXCR mRNA in various tumor tissues and normal ovarian tissues (based on TPM values). T represents the tumor group, and N represents the normal control group. High-expressed genes on the chromosome are marked with a red line, while low-expressed genes are marked with a green line. The red font indicates a difference. (b) The expression of CXCRs in normal ovarian tissue was compared with that of the ovarian tumor tissues, and the ^∗^ marked in red indicates significant differences.

**Figure 3:**
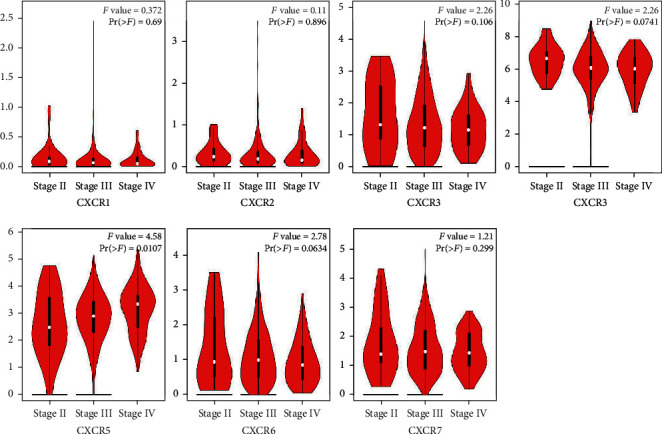
Gene expression profiling interactive analysis (GEPIA) of CXCRs in ovarian cancer patients at different tumor stages. The black and white piano keys represent the expression levels of CXCRs at stages II, III, and IV. The Pr value is marked in the upper right corner of the plot, and Pr < 0.05 indicates that the difference is statistically significant. CXCR expression at stage I has no corresponding results.

GEPIA is an interactive web application based on TCGA and GTEx large genome database data mining and gene function analysis and is an important data analysis tool. The online tool GEPIA database was used to compare mRNA expression levels of CXCR subfamily (CXCR1-CXCR7) in normal ovarian and ovarian tumor tissues and investigate their relationship with clinical stage. The mRNA expression levels of CXCR3 and CXCR4 in ovarian tumor tissues were significantly higher than those in normal ovarian tissues (Figure 2(a)). However, there was no significant difference in other family members of CXCR1, CXCR2, CXCR5, CXCR6, and CXCR7. The expression level of CXCR4 in ovarian tumor tissues was significantly higher than that of CXCR3 (Figure 2(b)). In the analysis of the relationship between CXCR subfamily and clinical stage, it was found that CXCR5 was positively correlated with the clinical stage of ovarian cancer (Figure 3), that is, the higher the expression, the more serious the clinical stage, suggesting that CXCR5 may be closely related to the metastasis of ovarian cancer.

**Figure 4:**
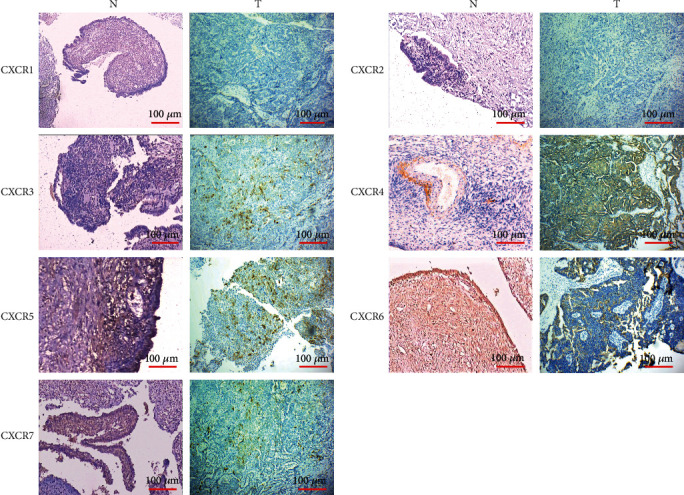
Immunohistochemistry results of CXCRs in epithelial ovarian cancer tissues. (a) CXCR1 is negative in ovarian cancer tissue and control; (b) CXCR2 is negative in ovarian cancer tissue and control; (c) CXCR3 is positive in ovarian cancer tissue and negative in control; (d) CXCR4 is strongly positive in ovarian cancer tissue and weakly positive in control; (e) CXCR5 is medium positive in ovarian cancer tissue and control; (f) CXCR6 is strongly positive in ovarian cancer tissue and control; (g) CXCR7 is positive in ovarian cancer tissue and weakly positive in control. Scale bar: 100 *μ*m.

Immunohistochemistry was used to detect the expression of CXCR protein in ovarian cancer tissues (Figure 4). The results showed that CXCRs were expressed in both cell membrane and cytoplasm of ovarian cancer tissues, among which CXCR4 expressions were the highest, and CXCR3, CXCR5, CXCR6, and CXCR7 were expressed in different degrees. The expression levels of CXCR3 and CXCR4 in ovarian tissues were obviously higher than those in control. These results were in consistent with the bioinformatics analysis.

### 3.3. Relationship between CXCR Expression and OS, PFS, and PPS in Patients with Ovarian Cancer

**Figure 5:**
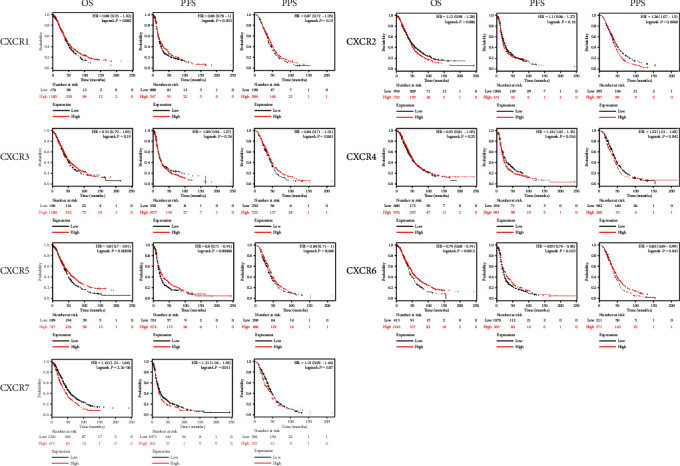
The prognostic value of mRNA level of CXCR factors in ovarian cancer patients (Kaplan-Meier plotter). The HR and logrank *P* values are indicated at the up right corner of the plots. Logrank *P* < 0.05 indicates that the difference is statistically significant.

Kaplan-Meier plotter online analysis tool was used to plot the OS curves of CXCR family members in 1657 ovarian cancer patients, the PFS curves in 1435 patients, and the PPS curves in 782 patients. The difference in survival was analyzed by logrank test. The results showed that the OS of the CXCR7 mRNA high expression group was lower than that of the CXCR7 low expression group at all time points. However, the OS of the CXCR5/6 mRNA high expression group was higher than that of the CXCR5/6 low expression group (Figure 5). This suggests that CXCR5/6 is an important protective factor, while CXCR7 is a risk factor of ovarian cancer. It was also found that CXCR4/7 mRNA high expression and CXCR5/6 mRNA low expression were associated with PFS shortening. Notably, high expression of CXCR2/4 and low expression of CXCR5/6 were closely related to PPS shortening. The mRNA of CXCR 2/4/5/6/7 can be used as indicators for predicting ovarian cancer progression.

### 3.4. Variation, Correlation, and Interaction Network of CXC Receptor Family in Ovarian Cancer Tissues

**Figure 6:**
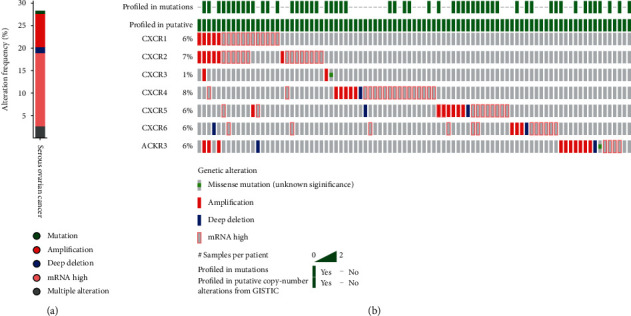
CXCR gene expression and mutation analysis in advanced ovarian cancer (cBioPortal). (a) CXCR gene mutation in ovarian cancer; (b) the detailed informed on the gene mutation of CXCR1-CRCX7. This suggests that GNB1, PTK2, MAPK1, PIK3CA, GNB4, GNA11, KNG1, and ARNT are closely related to CXC receptor family.

The cBioPortal was used to analyze the variation, correlation, and interaction gene network in TCGA database. The results showed that in the 586 ovarian malignant tumor samples, nearly 30% had mutations in the CXCR family, of which about 1% had mutations, less than 2% had deletions, and about 8% had amplification (Figures 6(a) and 6(b)). There was more than 17% of the patients had increased expression of CXCRs (Figure 6(a)).

**Figure 7:**
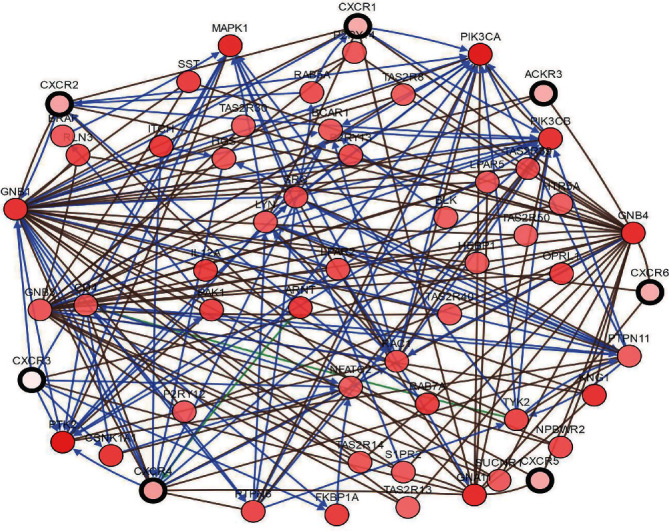
The interaction network of CXCR family proteins (cBioPortal). Color annotations highlight groups of regulatory nodes that may include the same pathway class or biological process.

Protein interaction network analysis revealed that GNB1, PTK2, MAPK1, PIK3CA, GNB4, GNA11, KNG1, and ARNT proteins were closely related to the CXC receptor family (Figure 7). These results indicate that changes in the molecular spectrum of the CXCR family contribute to the development of ovarian cancer.

### 3.5. GO and KEGG Pathway Enrichment Analyses of CXC Receptor Family

**Figure 8:**
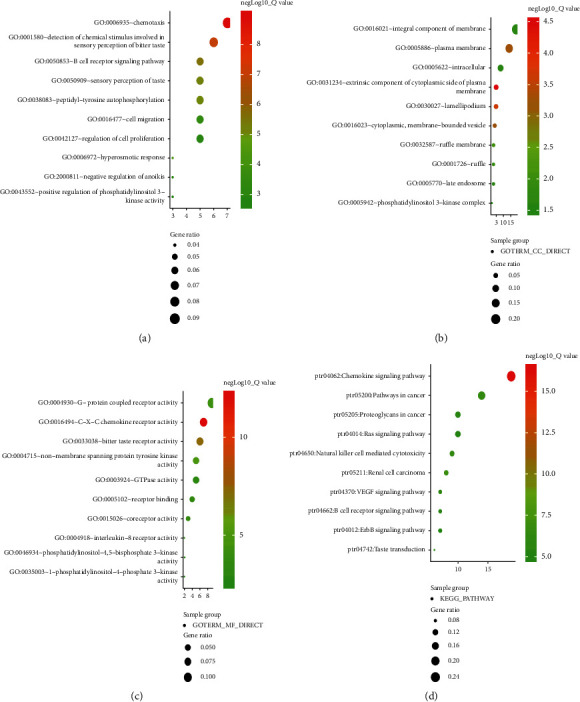
The functions of CXCRs predicted by analysis of Gene Ontology (GO) and KEGG by DAVID. (a) Enrichment analysis of the top 10 GO terms in the biological process; (b) enrichment analysis of 10 GO terms in molecular function; (c) enrichment analysis of the top 10 GO terms in cytology component; (d) enrichment analysis of the top 10 KEGG pathways.

GO analysis of the CXCR family was performed using the DAVID online tool. GO analysis mainly includes molecular function, biological process, and cell composition. There were 17 enriched biological processes, mainly including CXC chemokine receptor, nontransmembrane protein tyrosine kinase, G-protein-coupled receptor, ATP, guanosine triphosphate, and signal sensor activity (Figure 8(a)). The 10 enriched molecular functions included cell proliferation, migration, chemotaxis, and peptidyl-tyrosine autophosphorylation (Figure 8(b)). There were 21 enriched cytological components, which were mainly related to the exogenous components of the cytoplasmic side of the plasma membrane, flaky pseudopods, cell membranes and wrinkles, lysosomes, actin cytoskeleton, phosphatidylinositol 3-kinase complex, and so on (Figure 8(c)). Through the analysis of the above functions, the cell localization, geometric distribution, and functional categories of the CXCR family were further understood.

KEGG analysis showed that 72 pathways in ovarian cancer were associated with CXCR, and the top 10 pathways (Figure 8(d)) were ptr04062: chemokine signaling pathway; ptr05211: renal cell carcinoma; ptr04650: natural killer cell-mediated cytotoxicity; ptr05200: pathway in cancer; ptr04370: VEGF signaling pathway; ptr05205: proteoglycan in cancer; ptr04662: B cell receptor signaling pathway; ptr04742: taste transfer; ptr04014: Ras signaling pathway; and ptr04012: ErbB signaling pathway. These pathways are closely related to tumor metastasis, invasion, and drug resistance. These results can help to understand the potential molecular mechanism of CXCRs in the development of ovarian cancer and provide a theoretical basis for clinical targeted therapy.

## 4. Discussion

Metastasis, invasion, recurrence, and drug resistance are the main factors restricting the prolongation of survival in patients with ovarian cancer. The CXCR family plays an important role in the occurrence, metastasis, and prognosis of various tumors, but its mechanism is complex. For the first time, this study used bioinformatics tools to investigate the relationship between CXCR family and the development and prognosis of ovarian cancer. Our results demonstrate that CXC receptor family members may be used as new therapeutic targets and predicting markers of ovarian cancer.

CXCR1/CXCR2 are specific receptors for CXCL8, and their sequence similarity is about 75%. They have synergistic effects [23]. The ONCOMINE online database was used to compare the expression of CXCR1/CXCR2 mRNA in normal ovarian and ovarian cancer tissues. There was no significant difference between the two receptors. They were not related to clinical stage of ovarian cancer. However, their levels were increased in different pathological types of ovarian tumor tissues [24]. A large number of studies have shown that the ligand IL-8 of CXCR1/CXCR2 is abnormally increased in the plasma of patients with ovarian malignant tumors and is positively correlated with the clinical stage and pathological type of epithelial ovarian tumor (EOC) [25, 26]. There is also an abnormal increase in CXCL8 level in the ascites of EOC patients with abdominal metastases [27]. Moreover, our data showed that the high expression of CXCR2 indicated a shortened PPS.

The role of CXCR3 in tumors is unclear, and it is a controversial chemokine receptor. It is mainly expressed on the surface of activated immune cells such as T cells, B cells, and natural killer cells and binds to specific ligands (CXCL9, CXCL10, and CXCL11). On the one hand, CXCR3 binds to its ligand to activate the immune effector and inhibit tumor growth and on the other hand promote tumor growth and metastasis. Studies have shown that high CXCR3 expression in tumor tissues suggests poor prognosis in breast cancer [28], colorectal cancer [29], kidney cancer [30], and ovarian cancer, and inhibition of CXCR3 expression can reduce the production of ovarian cancer ascites [31]. In this study, it was found that CXCR3 was highly expressed in ovarian tumor tissues and expressed in different degrees in different types of ovarian cancer, but it was not related to clinical stage. For the survival analysis, CXCR3 expression was not associated with OS, PFS, and PPS, which may be related to its dual role or its splice variant type [32].

CXCR4 mRNA is one of the most expressed members of the family in ovarian malignancies [33]. Overexpression of CXCR4 can promote the proliferation and invasion of ovarian cancer cells, while the inhibitor AMD3100 and shRNA silencing CXCR4 can inhibit epithelial-mesenchymal transition, thereby inhibiting tumor proliferation, metastasis, and cell activation [34]. miR-9 [35] inhibits the expression of extracellular signal-regulating kinases ERK1, ERK2, and MMP-9 by inhibiting the CXCR4-CXCL12 signaling pathway, and the long-chain noncoding RNA LSLINCT5 [36] also plays an important role in ovarian cancer metastasis by regulating the CXCR4-CXCL12 signaling pathway. In this study, although the overexpression of CXCR4 was not significantly correlated with the OS, it was closely related to PFS and PPS. The higher the expression of CXCR4 was, the shorter the PFS and PPS. Thus, overexpression of CXCR4 is an important risk factor for advanced ovarian cancer. Studies have shown that CXCR4 is associated with drug resistance [37]. Cisplatin can increase the expression of CXCR4, which can promote the proliferation of cancer stem cells and enhance drug resistance, forming a vicious circle [37]. This indicates that the activation of CXCR4-CXCL12 pathway can cause a series of pathological changes such as ovarian tumor metastasis, tumor cell activation, angiogenesis, and drug resistance. Therefore, this pathway may be a potential target for the treatment of EOC patients and may be closely related to ovarian cancer drug resistance.

CXCR5, also known as Burkitt's lymphoma receptor 1, is abnormally increased in a variety of tumors such as gastric cancer [38], breast cancer [39, 40], intestinal cancer [41], prostate cancer [42], malignant neuroblastoma [43], and lung cancer [44], and it is significantly associated with poor prognosis. However, the relationship between CXCR5 expression and ovarian cancer has rarely been reported. This study found that there was no significant difference in the expression of CXCR5 between normal ovarian and ovarian cancer tissues. CXCR5 was the only CXC family member that not only related to the clinical stage of ovarian cancer, but also negatively correlated to the OS, PFS, and PPS of ovarian cancer patients. It may be an important indicator for predicting metastasis, recurrence, and drug resistance of ovarian cancer and may be used for diagnosis, therapy, and prognostic of ovarian cancer. However, it is puzzling that the higher the expression of CXCR5 is, the more advanced the clinical stage, suggesting it plays a role of protooncogene. However, it also plays the role of a tumor suppressor gene in the relationship with prognosis. This controversy is the focus of our future study.

The expression of CXCR6 in ovarian cancer tissues is higher than that in adjacent tissues and is positively correlated with the expression of TNF-*α*. Macrophages promote the migration and invasion of ovarian cancer by binding to its unique ligand CXCL16 to activate PI3K/Akt signaling pathway [35]. The expression of CXCR6 in epithelial ovarian cancer is significantly higher than that in normal ovarian and benign tissues. Studies have shown that the expression of CXCR6 is associated with lymph node metastasis [36], and the same conclusions have been obtained in cell experiments [45]. Ovarian cancer cell lines OVCAR-3 and SKOV-3 with high expression of CXCR6 have higher migration and invasion abilities. Interestingly, in the early stage of ovarian cancer metastasis, mesothelial cells promote peritoneal mesothelial fibroblast transformation by activating CXCR6, thereby promoting ovarian cancer proliferation and peritoneal metastasis [46]. However, another study showed that there was no difference in the expression level of CXCR6 between ovarian tumor primary lesions and omental metastases lesions [47]. Study has shown that the monoclonal antibody targeting of CXCR6 can increase the sensitivity of docetaxel [48], indicating that overexpression of CXCR6 may be as a target to improve drug resistance. In addition, overexpression of CXCR6 is associated with poor prognosis in prostate cancer [49], gastric cancer [50], and renal cancer [51], especially prostate cancer CXCR6 which is an independent predictor of poor prognosis, and its overexpression is a risk factor [49]. However, this study obtained an opposite conclusion that the overexpression of CXCR6 was associated with OS, PFS, and PPS of ovarian cancer. Further research is needed to verify this.

CXCR7 belongs to the non-G-protein-coupled receptor [52], which not only competitively binds to the ligand CXCL12, but also binds to CXCL12 up to 10-fold more than CXCR4. Binding of the CXCR7 receptor to the ligand CXCL12 activates the p38 MAPK pathway to promote MMP-9 expression, thereby enhancing ovarian cancer cell invasion [53]. However, there are different results showing that CXCR4/CXCL12 axis promotes EMT and is a potential target of ovarian cancer progression. CXCR7 does not play a key role in EMT, but CXCL12/CXCR4 axis is a potential target for preventing ovarian cancer progression [34]. In addition to this, CXCR7 can also bind to CXCL11 [54] and CCL19 [55]. The CCL19/CXCR7 axis activates the AKT and ERK pathways and downregulates the expression of E-cadherin. CXCR7 overexpression not only significantly enhances histone modification and transcription, but also indirectly induces the expression of mesenchymal markers such as SNAI1, SNAI2, and CDH2 to promote migration and invasion of ovarian cancer cells [58]. This study found that although there was no significant difference in CXCR7 expression between ovarian and normal ovarian tissues and CXCR7 expression was not associated with clinical stage, CXCR7 was negatively correlated with OS and PFS, whereas CXCR4 was not associated with OS but associated only with PFS and PPS. The combination of CXCR7 and CXCR4 is a very promising indicator for predicting the occurrence, progression, and prognosis of ovarian cancer. In the CXCR7-CXCR4/CXCL12 axis, whether CXCR4 and CXCR7 are a competitive inhibition relationship or have a synergistic effect is still controversial.

## 5. Conclusions

In conclusion, the expression and prognostic value of the CXC receptor family in ovarian cancer was analyzed, and the changes in the molecular profile of CXCs in patients with ovarian cancer were further understood. The results of this study indicate that CXCR3/4/7 are potential targets for the treatment of ovarian cancer, and CXCR2/4/5/6/7 are new markers for the prognosis of ovarian cancer. Among them, CXCR5 and CXCR6 have been found to play a role of tumor suppressor genes in the prognosis, which is controversial. Further studies are needed to validate these results.

## Figures and Tables

**Table 1:** CXCR mRNA expression difference in ovarian cancer and ovarian tissues (ONCOMINE database).

CXC	Types of ovarian cancer vs. ovarian	*P* value	*t*-test	Fold change	Ref.
CXCR1	Ovarian cancer vs. normal	NA	NA	NA	NA
	Ovarian adenocarcinoma type vs. ovarian endometrioid adenocarcinoma	2.29*E*-5	6.178	2.348	[13]
	Ovarian carcinoma type vs. ovarian adenocarcinoma	8.71*E*-4	5.043	1.825	[11]
CXCR2	Ovarian cancer vs. normal	NA	NA	NA	NA
	Cancer type vs. ovarian cancer	2.69*E*-4	4.050	2.477	[15]
	Ovarian adenocarcinoma type vs. ovarian endometrioid adenocarcinoma	0.020	2.084	1.628	[16]
	Ovarian adenocarcinoma type vs. ovarian mucinous adenocarcinoma	0.014	2.476	2.168	[16]
CXCR3	Ovarian serous surface papillary carcinoma vs. normal	0.014	2.333	1.943	[22]
	Ovarian carcinoma type vs. ovarian adenocarcinoma	0.022	3.157	1.683	[17]
	Cancer type vs. ovarian cancer	0.005	2.853	2.623	[11]
	Cancer type vs. ovarian cancer	0.010	2.416	2.824	[18]
CXCR4	Ovarian serous surface papillary carcinoma vs. normal	2.44*E*-19	22.51	306.41	[15]
	Ovarian serous adenocarcinoma vs. normal	0.012	2.958	3.156	[19]
	Ovarian carcinoma vs. normal	3.05*E*-6	7.594	2.632	[21]
	Ovarian mucinous adenocarcinoma vs. normal	0.018	2.466	1.9	[22]
	Ovarian serous adenocarcinoma vs. normal	0.011	2.632	2.086	[22]
	Ovarian serous adenocarcinoma vs. normal	0.021	2.256	1.818	[23]
	Cancer type vs. ovarian cancer	0.010	2.622	2.403	[24]
	Ovarian adenocarcinoma type vs. ovarian serous adenocarcinoma	0.002	2.977	1.720	[25]
	Cancer type vs. ovarian carcinoma	0.002	3.092	2.041	[26]
	Cancer type vs. ovarian cancer	0.018	2.164	2.340	[27]
	Cancer type vs. ovarian carcinoma	2.94*E*-7	5.484	2.355	[28]
	Cancer type vs. ovarian carcinoma	2.49*E*-6	5.976	2.566	[29]
CXCR5	Ovarian cancer vs. normal	NA	NA	NA	NA
	Cancer type vs. ovarian cancer	NA	NA	NA	NA
CXCR6	Ovarian cancer vs. normal	NA	NA	NA	NA
	Cancer type vs. ovarian cancer	NA	NA	NA	NA
CXCR7	Ovarian serous surface papillary carcinoma vs. normal	0.046	1.751	1.698	[15]
	Ovarian cancer vs. ovarian carcinoma	1.20*E*-6	5.598	2.027	[29]
	Ovarian cancer vs. ovarian carcinoma	2.74*E*-5	4.292	1.937	[28]

## Data Availability

The data that support the findings of this study are available on request from the corresponding author.
